# The Sick and Infirm

**Published:** 1907-11-30

**Authors:** 


### HOSPITAL ADMINISTRATION.

### CONSTRUCTION AND ECONOMICS.

### THE SICK AND INFIRM.

In an interesting article published on November 21 a correspondent gives a layman's view in the *Times* of certain matters which intimately affect the administration of the hospitals throughout the British Islands, especially in large cities. He very rightly lays stress upon the new spirit which he describes as a creative force at work in the present day, which directly encourages managers of institutions to look beyond the individual position of the particular charity. It is within our recollection, and many instances of the same spirit are still to be met with in the hospital world, when it was practically impossible to find any hospital of importance, the managers of which did not show an inclination to approach all questions of reform from the point of view that, however many might be the evils existing elsewhere, in their particular institution the system was practically perfect. This spirit had much to do with the halting and imperfect methods which were formerly adopted with a view to introduce reform in various directions.

At the moment the absence of co-operation among the nurse-training schools leaves the field of nursing open to exploitation by any restless spirit whose ambition may lead its possessor to desire to be regarded as an authority on nursing. If the nurse-training schools would combine we should soon have a central examination body, which would speedily enable all nurses who were really competent, though they might from no fault of their own have failed to obtain a certificate of three years' training in one particular hospital, to show their capacity and to obtain a diploma which would give them the position which they may be entitled to from their knowledge and work. If this result was once secured, all the existing abuses which arise from the absence of a system to prevent incompetent women from acting as nurses would disappear. Further, the nursing world would be freed from the continuous agitation, which is kept alive by a few ambitions spirits, who have really little or no claim to speak with authority on nursing matters. All such attempts of individuals to arrogate to themselves the exclusive right to govern the nursing world would so be rendered abortive, and trained nurses would be protected from agitations which tend to make nurses so unpopular in this country.

There is some hope that this matter may, in due course, come under the notice of the authorities of the King's Fund, because at the present time certain hospitals do undoubtedly train a larger number of nurses, than they require for supplying attendants for the sick in the wards. The case of certain lying-in hospitals is very much to the point in this connection, for the number of women they sometimes receive for training in their schools is out of all proportion to the number of beds, and to the number of patients which have to be dealt with in the course of each year. In the same way, but to a less extent, the increase in the number of probationers admitted to training at some of the larger hospitals is undoubtedly beyond the immediate requirements of the individual institutions. If the present system is to continue, the cost of the training of such extra probationers may one day have to be provided from funds distinct from those subscribed for the relief of the sick in the hospitals.

Again, the *Times* correspondent properly commends the system by which King Edward's Hospital Fund has organised the personal visitation of hospitals by Visitors from the Fund. These Visitors are carefully chosen for their personal qualities and their knowledge of the subject, and they do their work under strict conditions of privacy, while care is taken to avoid anything approaching inquisitorial interference. The Visitors' duties are responsible, because their reports may be regarded as reliable evidence on which to base an opinion as to a particular hospital, its requirements and the purposes to which money may be applied. A further point which the correspondent commends is the attempt made by King Edward's Hospital Fund to bring about the amalgamation of groups of the smaller hospitals. The orthopædic hospitals have now been consolidated, and a serious effort is being made to combine all special hospitals which treat diseases of the throat, ear, and nose. There can be no doubt that one great orthopædic hospital, and one great throat, ear, and nose hospital, if they were to be organised in the most perfect manner, and to be administered on the best principles of modern hospital management, could not fail to effect an infinitely greater amount of good work at infinitely less cost, than can be accomplished by a number of relatively small institutions working independently. A number of such institutions necessitates the multiplication of office-holders and office expenses, most of which would be saved under the larger scheme. The only real difficulty in the way of amalgamation would appear to be the natural tendency to jealousy on the part of individual officers, and the fear that some of them might lose their appointments if one great hospital were to be substituted for half a dozen small ones.

Is it not possible, as a preliminary step, that the smaller hospitals belonging to each group, though continuing their work in the existing buildings, might be managed by one committee with one secretary and one office? Then, as the members of the medical staff retire in due course, arrangements might be made not to fill the vacancies thus created, and in time funds should accumulate for the building of a large central hospital of the very highest efficiency, whilst a portion, or the whole, of the existing buildings of the smaller institutions might be retained as receiving-stations and out-patient departments.

The correspondent of the *Times* deals with a statement by the Duke of Fife made 18 months ago, when he presided at an annual meeting of the King's Hospital Fund. The Duke said then the needs and necessities of the hospitals have grown enormously in the last 20 years, and if it were not for doubtful finance, sensational appeals, and every form of begging, many of them would be obliged to close their doors. We would venture to point out, however, that every year the instances of doubtful finance and sensational appeals grow happily less. The truth is that during the last 40 years, and especially during the last 20, through the persistent, continuous, and purposeful effort of a few men, who have devoted much time and thought to the subject, the public have been awakened to the importance of the voluntary hospitals as one of the greatest and most valuable planks in our modern social system. These efforts have resulted in a widening of the area of givers to an extent which has created a public opinion on the subject, and the trend of that opinion is to discourage sensational appeals. We believe, as we have often said, that those liberal givers who, at the present time, form the backbone of hospital finance under the voluntary system, have come to the decision not to give, largely, to any hospital, unless the appeals it issues are business documents, free from sensation and exaggeration. It would not be difficult to quote a number of instances in proof of this view, but anyone, who has any doubt on the subject, may find all the evidence he wants in support of it by a perusal of the article we published on Guy's Hospital in The Hospital of November 16, page 189. That article proves that a good cause, a business appeal, and quiet, steady, purposeful work can produce enormous sums of money for hospital purposes, at a cost, so small, as to be less than 5 per cent. of the total sum raised.

We venture, therefore, to say that whatever grounds there might have been for an opinion such as that expressed by the Duke of Fife, those grounds are growing less and less each year. This fact is the strongest possible proof of the great advance in efficiency which the voluntary hospitals have displayed in recent years.

